# Metronidazole, an Uncommon Cause of Dizziness and Ataxia in the Emergency Department: A Case Report

**DOI:** 10.5811/cpcem.2021.3.52046

**Published:** 2021-05-10

**Authors:** Mary E. Starrs, Onur M. Yenigun

**Affiliations:** Stanford University Medical Center, Department of Emergency Medicine, Palo Alto, California

**Keywords:** Metronidazole, case report, ataxia

## Abstract

**Introduction:**

Metronidazole, a nitroimidazole antibiotic, is a well-known antibacterial and antiprotozoal agent that is generally well tolerated without many serious side effects. Most adverse reactions affect the gastrointestinal or genitourinary system, but the central nervous system may also be afflicted. In addition to headache and dizziness, cerebellar dysfunction can occur with metronidazole use.

**Case Report:**

We discuss the clinical presentation and imaging findings of metronidazole-induced encephalopathy in a 12-year-old male. The patient had a history of Crohn’s disease and chronic *Clostridium difficile* infection for which he had received metronidazole for approximately 75 days prior to arrival to a local emergency department (ED). He presented with five days of progressive vertigo, nausea, vomiting, and ataxia. Subsequent magnetic resonance imaging showed symmetric hyperintense dentate nuclei lesions, characteristic of metronidazole-induced encephalopathy. The patient’s symptoms improved rapidly after cessation of metronidazole, and his symptoms had completely resolved by discharge on hospital day two.

**Conclusion:**

Metronidazole-induced encephalopathy is a rare cause of vertigo and ataxia that can lead to permanent sequela if not identified and treated promptly. Thus, it is important for physicians to keep this diagnosis in mind when evaluating patients on metronidazole who present to the ED with new neurologic complaints.

## INTRODUCTION

Metronidazole has been used to fight various bacterial and protozoal infections globally for more than 50 years. The majority of adverse reactions are minor, and the drug is generally well tolerated. The most common side effects reported include nausea, headache, and metallic taste. The central nervous system (CNS) is not spared, however, as metronidazole easily penetrates the blood-brain barrier and can accumulate, leading to neurologic toxicity. In a systemic review of metronidazole-induced encephalopathy (MIE), Sorensen et al found that most patients with CNS involvement present with dysarthria (63%), followed by gait instability (55%). Other clinical findings include limb dyscoordination and vertigo (53% and 18%, respectively).^1^

These findings suggest that the cerebellum serves as a primary site of metronidazole accumulation, as this region of the CNS is the major regulator of motor movement and coordination. Magnetic resonance imaging (MRI) data of patients with MIE support this finding. Specifically, on T2-weighted and fluid-attenuated inversion recovery sequences (FLAIR), symmetric hyperintense dentate nuclei lesions were the most common abnormal findings (90%).^2^ The following report describes a patient with MIE who was ultimately diagnosed and treated after MRI.

## CASE REPORT

A 12-year-old male with Crohn’s disease on prednisone, chronic granulomatous disease, as well as chronic *Clostridium difficile* infection on metronidazole presented to the emergency department (ED) with a chief complaint of dizziness. The patient and his mother reported that he had experienced several intermittent episodes of vertigo, sometimes associated with movement, as well as nausea and vomiting over the prior five days. Additionally, over the previous three days, the patient had become progressively more unstable with ambulation and required assistance to get to and from the bathroom. He otherwise denied headache, ear pain, recent infection, or other focal neurologic deficits. In consultation with his outpatient providers, the patient’s prednisone dose was increased from a baseline of 5 milligrams (mg) to 10 mg, and then up to 15 mg after one and two days of symptoms, respectively, due to concern for adrenal insufficiency.

Upon examination in the ED, the patient was noted to have normal vital signs for age, as well as normal cardiopulmonary and abdominal examinations. A detailed neurologic exam was notable for an unsteady, wide-based gait, at which time the patient reported a sensation of significant imbalance and intermittent vertigo. The remainder of his neurologic exam, including cranial nerves, sensation, motor strength, Romberg’s test, and heel-to-shin as well as finger-nose-finger testing, was normal. Given that these findings were concerning for a cerebellar pathology, neurology was consulted and the patient was admitted to the general pediatric floor for further workup.

An MRI of the brain obtained the following morning demonstrated T2/FLAIR hyperintensity in the dentate nuclei of the cerebellum. These findings were consistent with metronidazole toxicity as described above.^2^ The patient’s metronidazole was subsequently stopped in the afternoon. The following morning he had a significant improvement in symptoms. He had no further vertigo, vomiting, or gait unsteadiness and was discharged the following day.

## DISCUSSION

Metronidazole-induced encephalopathy is a rare adverse effect of prolonged metronidazole use. In the aforementioned systematic review of 136 cases, the duration of treatment with the antibiotic before the onset of CNS dysfunction varied from a lower quartile of 14 days to an upper quartile of 52.5 days, with an average of 47.2 days.^1^ Our patient had been consistently taking metronidazole for approximately 75 days before the development of symptoms. A leading theory is that the pathogenesis may be secondary to axonal swelling caused by vasogenic edema, possibly due to impairment in vitamin B1 activity given metronidazole’s conversion to a thiamine analog in vivo.^3^

It has been shown in previous studies that metronidazole-induced neurologic symptoms often improve within a few days after drug withdrawal.^4^ In the systematic review by Sorenson et al of 136 patients, 119 patients had either improvement or complete resolution of CNS symptoms after stopping metronidazole. Twelve of 136 patients had unfavorable outcomes including death or persisting neurologic deficits. Of these, almost all had previous premorbid conditions including organ failure or cancer. Interestingly, six of these surviving patients had cerebral white matter lesions on MRI in addition to the dentate nuclei lesions, suggesting that these lesions may be associated with worse prognosis (Image).^1^ Other studies have shown that steroid treatment may hasten recovery.^5^ It is believed that the immunosuppressive effects of steroids serve to decrease the vasogenic edema that may contribute to metronidazole’s neurotoxicity as discussed above.^4^ Our patient’s neurologic symptoms resolved completely within 24 hours of stopping the metronidazole. This rapid recovery may have been in part attributable to the recent increase in his prednisone dosage.

**CPC-EM Capsule**

What do we already know about this clinical entity?

*Metronidazole is a commonly used antibiotic often associated with minor side effects affecting the gastrointestinal system, such as nausea and diarrhea.*

What makes this presentation of disease reportable?

*Metronidazole toxicity is an uncommon but often reversible cause of new neurologic symptoms in patients presenting to the emergency department with these complaints.*

What is the major learning point?

*Prolonged metronidazole use has been associated with new neurologic symptoms and characteristic magnetic resonance imaging findings that often are reversible with discontinuation of use.*

How might this improve emergency medicine practice?

*Broadening the differential when evaluating a patient with new neurologic complaints will aid in early diagnosis of metronidazole toxicity.*

## CONCLUSION

This case highlights the importance of thorough history-taking, including prescribed medications, when evaluating symptoms of vertigo and cerebellar dysfunction. Furthermore, we highlight key, specific MRI findings that are suggestive of metronidazole-induced encephalopathy. Due to its rarity, MIE diagnosis may be delayed. However, since the first case of MIE was reported in 1978, there have been at least 135 documented cases. Of these, 50 have been reported in the last five years, thus emphasizing the increasing prevalence of the condition.^1^ Additionally, the importance of diagnosis is crucial as some patients may develop persistent neurologic deficits or even death.^5^,^6^ As the ED is often the first place of contact for patients with new neurological deficits, it is important the emergency physician keep this rare, but often reversible, diagnosis in mind when evaluating a patient with a new neurologic compliant.

## Figures and Tables

**Image:**
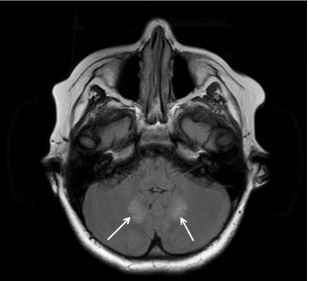
T2 and fluid-attenuation inversion recovery sequence magnetic resonance imaging of brain showing symmetric hyperintense dentate nuclei lesions that are characteristic of metronidazole-induced encephalopathy.
